# New Method of Giving Chloroform

**Published:** 1862-05

**Authors:** 


New Method of Giving Chloroform.—At a recent meeting of the Obstetrical Society, Dr. Simpson stated that he now preferred to administer chloroform by laying a single layer of handkerchief over the face and letting the chloroform fall on it drop by drop. The advantages are said to be: that there is less danger to the patient from the smaller quantity applied at a time; that anæsthesia is more speedily produced, and that the quantity of chloroform required is less. Various gentlemen confirmed the value of this process, and Dr. Young in particular stated that he had kept a patient narcotized for ten hours with two ounces and a half of chloroform.—*Brit. Med. Journ.; Amer. Med. Times.*

Action of Arsenic.—In a paper read before the Royal Medical and Chirurgical Society of London, Prof. G. Harley, speaking of his experiments in reference to slow or chronic poisoning, gave the following conclusions in regard to the direct action of arsenic on the blood. Arsenic has a specific action on the digestive canal, and this action is manifested irrespectively of the mode of administration. The direct contact-action with the mucous membrane is slight in comparison to the influence exerted through the blood. The symptoms manifested during life, as well as the morbid changes found after death, differ very materially in the acute and chronic forms of poisoning. While in the former the morbid changes are most marked at the cordiac end of the stomach, in the chronic form they are most visible toward the pyloric extremity. The more gradual the poisoning, the more manifest is the action of the poison on the intestines, and the less visible are its effects on the stomach. Death may occur from arsenic so rapidly that no apparent structural change has time to take place. The immunity from symptoms of poisoning enjoyed by arsenic-eaters, most probably arises from their taking the substance in a solid form, so that but a very small portion of what is swallowed enters the circulation. The beneficial effects of small doses of arsenic are due to the power it possesses of diminishing tissue-change by its peculiar action on the blood. The prejudicial effects of arsenic, when taken in excess, are due to its destroying the property possessed by the constituents of the blood of combining with oxygen and thereby becoming fitted for the purposes of nutrition.—*Boston Med. and Surg. Journal.*

